# Retrospective Analysis of the Survival Benefit of Induction Chemotherapy in Stage IVa-b Nasopharyngeal Carcinoma

**DOI:** 10.1371/journal.pone.0160758

**Published:** 2016-08-10

**Authors:** Xiao-Wen Lan, Xue-Bin Zou, Yao Xiao, Jie Tang, Pu-Yun OuYang, Zhen Su, Fang-Yun Xie

**Affiliations:** 1 Department of Radiation Oncology, Sun Yat-sen University Cancer Center, State Key Laboratory of Oncology in South China, Guangzhou, Guangdong Province, People's Republic of China; 2 Department of Ultrasound, Sun Yat-sen University Cancer Center, State Key Laboratory of Oncology in South China, Guangzhou, Guangdong Province, People's Republic of China; University of Nebraska Medical Center, UNITED STATES

## Abstract

**Purpose:**

The value of adding induction chemotherapy to chemoradiotherapy in locoregionally advanced nasopharyngeal carcinoma (LA-NPC) remains controversial, yet high-risk patients with LA-NPC have poor outcomes after chemoradiotherapy. We aimed to assess the survival benefits of induction chemotherapy in stage IVa-b NPC.

**Patients and Methods:**

A total of 602 patients with stage IVa-b NPC treated with intensity-modulated radiation therapy (IMRT) and concurrent chemotherapy with or without induction chemotherapy were retrospectively analyzed. Overall survival (OS), locoregional relapse-free survival (LRFS), distant metastasis-free survival (DMFS) and progression-free survival (PFS) were evaluated using the Kaplan-Meier method, log-rank test and Cox regression analysis.

**Results:**

In univariate analysis, 5-year OS was 83.2% for induction chemotherapy plus concurrent chemotherapy and 74.8% for concurrent chemotherapy alone, corresponding to an absolute risk reduction of 8.4% (*P* = 0.022). Compared to concurrent chemotherapy alone, addition of induction chemotherapy improved 5-year DMFS (83.2% vs. 74.4%, *P* = 0.018) but not 5-year LRFS (83.7% vs. 83.0%, *P* = 0.848) or PFS (71.9% vs. 66.0%, *P* = 0.12). Age, T category, N category, chemotherapy strategy and clinical stage were associated with 5-year OS (*P* = 0.017, *P* = 0.031, *P* = 0.007, *P* = 0.022, *P* = 0.001, respectively). In multivariate analysis, induction chemotherapy plus concurrent chemotherapy was an independent favorable prognostic factor for OS (HR, 0.62; 95% CI, 0.43–0.90, *P* = 0.012) and DMFS (HR, 0.57; 95% CI, 0.38–0.83, *P* = 0.004). In subgroup analysis, induction chemotherapy significantly improved 5-year DMFS in stage IVa (86.8% vs. 77.3%, *P* = 0.008), but provided no significant benefit in stage IVb.

**Conclusions:**

In patients with stage IVa-b NPC treated with IMRT, addition of induction chemotherapy to concurrent chemotherapy significantly improved 5-year OS and 5-year DMFS. This study provides a basis for selection of high risk patients in future clinical therapeutic trials.

## Introduction

Nasopharyngeal carcinoma (NPC) is one of the most common malignant tumor types in southern China, with high yearly incidences of between 15 and 50 cases per 100,000 reported [1–2]. Radiotherapy is the primary and only curative treatment for locally and regionally confined NPC. In centers where modern radiation technology is available, intensity-modulated radiation therapy (IMRT) is preferred over conventional techniques as it confers improved disease control and reduces the doses to the normal tissues [3–5].

Several clinical trials have confirmed chemoradiotherapy improves disease control and survival in locoregionally advanced nasopharyngeal carcinoma (LA-NPC), and this strategy has been established as the standard treatment [6–12]. However, controversy currently exists regarding the evidence supporting administration of induction chemotherapy to patients with LA-NPC. In a randomized phase 2 study, Hui et al. [13] showed 3-year progression-free survival (PFS) and overall survival (OS) differed significantly between induction chemotherapy plus chemoradiotherapy group and chemoradiotherapy alone group (88% vs. 94% and 60% vs. 68%, respectively). However, Fountzilas et al. [14] and Tan et al. [15] reported the addition of induction chemotherapy to chemoradiotherapy provided no significant survival benefit in LA-NPC.

Subgroup analysis of a chemoradiotherapy trial reported patients with high-risk features (N3, T4N2 disease or bulky nodal metastases) had poorer outcomes after chemoradiotherapy [16]. Therefore, it is important to evaluate whether the addition of induction chemotherapy provides a survival benefit in high-risk subgroups of patients with LA-NPC, such as patients with an advanced T category or N3 disease. In this study, we retrospectively assessed 602 patients with stage IVa-b NPC treated with IMRT plus concurrent chemotherapy with or without induction chemotherapy to further assess the value of induction chemotherapy in LA-NPC.

## Patients and Methods

### Patients

We reviewed the medical records of 602 patients with stage IVa-b NPC treated with IMRT plus concurrent chemotherapy (CC) with or without induction chemotherapy (IC) at Sun Yat-sen University Cancer Center between January 6, 2005 and February 22, 2012. This retrospective study was approved by the Institutional Review Board of Sun Yat-sen University Cancer Center. The requirement for written consent was waived.

### Pretreatment evaluation

The pretreatment patient evaluation included a complete medical history, physical and neurologic examinations, hematological studies and biochemical profiles. All patients underwent fiberoptic nasopharyngoscopy with biopsy, magnetic resonance imaging (MRI) of the nasopharynx and neck, chest radiography, abdominal sonography, and a whole body bone scan using single photon emission computed tomography (SPECT) or positron emission tomography computed tomography (PET/CT). All patients were restaged according to the seventh edition of the International Union against Cancer/American Joint Committee on Cancer (UICC/AJCC) staging system for NPC [17].

### Radiation therapy

All patients were treated using IMRT. Details of the technique have been reported previously [18–20]. The prescribed dose was 68–70 Gy to the planning target volume (PTV) of the primary tumor, 60–68 Gy to the PTV of the cervical lymph nodes, 60 Gy or greater to the PTV of CTV-1 (i.e. high-risk regions) and 54–56 Gy to the PTV of CTV-2 (i.e. low-risk regions and neck nodal regions) over 30–33 fractions. Radiotherapy was delivered over one fraction daily, 5 days per week.

### Chemotherapy

All patients received platinum-based chemotherapy. They received concurrent chemotherapy regimen of cisplatin or nedaplatin weekly [30 mg/m^2^ on days 1–3] or every three weeks during radiotherapy. The three weekly regimens were cisplatin or nedaplatin [80–100 mg/m^2^ on day 1], the PF regimen (nedaplatin [60–80 mg/m^2^ on day 1] and fluorouracil [600–1000 mg/m^2^ on days 1–5]), or the TP regimen (docetaxel [60–75 mg/m^2^ on day 1] and cisplatin [60–80 mg/m^2^ on day 1]). Induction chemotherapy consisted of the TP regimen (docetaxel [60–75 mg/m^2^ on day 1] and cisplatin [60–80 mg/m^2^ on day 1]), or TC regimen (paclitaxel [135 mg/ m^2^ on day 1] and carboplatin [area under the curve, five on day 1]), or TPF regimen (docetaxel [60 mg/m^2^ on day 1] and cisplatin [60 mg/m^2^ on day 1] and fluorouracil [600 mg/m^2^ on days 1–5]), or PF regimen (cisplatin or nedaplatin [60–80 mg/m^2^ on day 1] and fluorouracil [600–1000 mg/m^2^ on days 1–5]).

### Follow-up

The duration of patient follow-up was calculated from the first day of treatment to either the day of death or day of last follow up. Patients were examined every 3–6 months during the first 3 years and every 6–12 months thereafter until death. Locoregional recurrences were confirmed by endoscopy and MRI scans, and in doubtful cases, by biopsy. Distant metastases were diagnosed on the basis of clinical symptoms, physical examinations and imaging methods, including chest radiography, bone scans, magnetic resonance imaging (MRI) and abdominal sonography. Patients without recent examination records were followed-up via telephone calls.

### Statistical analysis

Statistical Product and Service Solution version 17.0 (IBM Corporation, Armonk, NY, USA) was used for statistical analyses. All events were measured from the date of treatment commencement. The following end points were assessed: overall survival (OS), distant metastasis-free survival (DMFS) and locoregional relapse-free survival (LRFS); these end-points were calculated from the date of first treatment to the date of death from any cause, first distant metastasis, or locoregional relapse, respectively, or until the date of last follow-up visit. Progression-free survival (PFS) was defined as the time from treatment to disease progression (distant metastasis or locoregional relapse), death due to any cause or last follow-up visit. Actuarial rates were calculated using the Kaplan-Meier method and differences were compared using the log-rank test. Multivariate analyses with the Cox proportional hazards model were used to identify significant independent prognostic factors and calculate hazard ratios (HRs). In multivariate analysis, the following parameters were included in the model as covariates for each analysis: gender; age (≤ 46 vs. > 46 years); World Health Organization (WHO) pathological type (types I, II, III); T category (T1, T2, T3, T4); N category (N0, N1, N2, N3); chemotherapy strategy (IC + CC and CC); clinical stage (IVa and IVb). Two-tailed *P*-values less than 0.05 were considered statistically significant.

## Results

### Patient characteristics

Among this cohort of 602 patients with stage IVa-b NPC treated with IMRT, 406 (67.4%) and 196 (32.6%) patients received concurrent chemotherapy with and without induction chemotherapy, respectively. The cohort included 472 males and 130 females (male:female ratio, 3.63:1). The median patient age was 46 years (range 13–78 years). Histologically, 93.7% (564/602) patients had World Health Organization (WHO) type III disease and 6.3% (38/602) had WHO type I or type II disease.

The patients who received induction chemotherapy plus concurrent chemotherapy and patients who received concurrent chemotherapy alone were not significantly different in terms of gender, age, histological type, T/N categories, and tumor factors. Table 1 summarizes the characteristics of all patients. The T and N category distributions for the 602 patients stratified by treatment are listed in Table 2.

**Table 1:** Characteristics of the 602 patients with stage IVa-b nasopharyngeal carcinoma treated with IMRT who underwent concurrent chemotherapy with or without induction chemotherapy.

Characteristic	IC + CC (*n* = 406)		CC (*n* = 196)		*P*-value*
	*n*	%	*n*	%	
Gender					0.723
Male	320	78.8	152	77.6	
Female	86	21.2	44	22.4	
Age(years)					0.623
≤ 46	222	54.7	103	52.6	
> 46	184	45.3	93	47.4	
WHO pathology^a^					0.733
Type I	5	1.2	1	0.5	
Type II	23	5.7	9	4.6	
Type III	378	93.1	186	94.9	
T category					0.599
T1	2	0.5	3	1.5	
T2	14	3.4	8	4.1	
T3	28	6.9	13	6.6	
T4	362	89.2	172	87.8	
N category					0.160
N0	52	12.8	34	17.3	
N1	184	45.3	96	49	
N2	93	22.9	40	20.4	
N3	77	19	26	13.3	
Clinical stage					0.082
IVa	329	81	170	86.7	
IVb	77	19	26	13.3	

Abbreviations: IMRT = intensity-modulated radiotherapy; IC = induction chemotherapy; CC = concurrent chemotherapy; WHO = World Health Organization.

^a^ Based on WHO histological type (1991): I—keratinizing squamous-cell carcinoma, II—differentiated non-keratinizing carcinoma, III—undifferentiated non-keratinizing carcinoma.

* *P*-values were calculated using chi-square test.

**Table 2:** T and N category distributions for the 602 patients with stage IVa-b nasopharyngeal carcinoma stratified by treatment group.

	*n* (%)	IC + CC	CC
		*n* (%)	*n* (%)
T1N3	5(0.8)	2 (0.5)	3(1.5)
T2N3	22 (3.7)	14 (3.5)	8 (4.1)
T3N3	41 (6.8)	28 (6.9)	13 (6.6)
T4N0	86 (14.3)	52 (12.8)	34 (17.3)
T4N1	280 (46.5)	184 (45.3)	96 (49.1)
T4N2	133 (22.1)	93 (22.9)	40 (20.4)
T4N3	35 (5.8)	33 (8.1)	2 (1.0)
total	602 (100)	406 (100)	196 (100)

Abbreviations: IC = induction chemotherapy; CC = concurrent chemotherapy.

### Survival analysis

**Fig 1:**
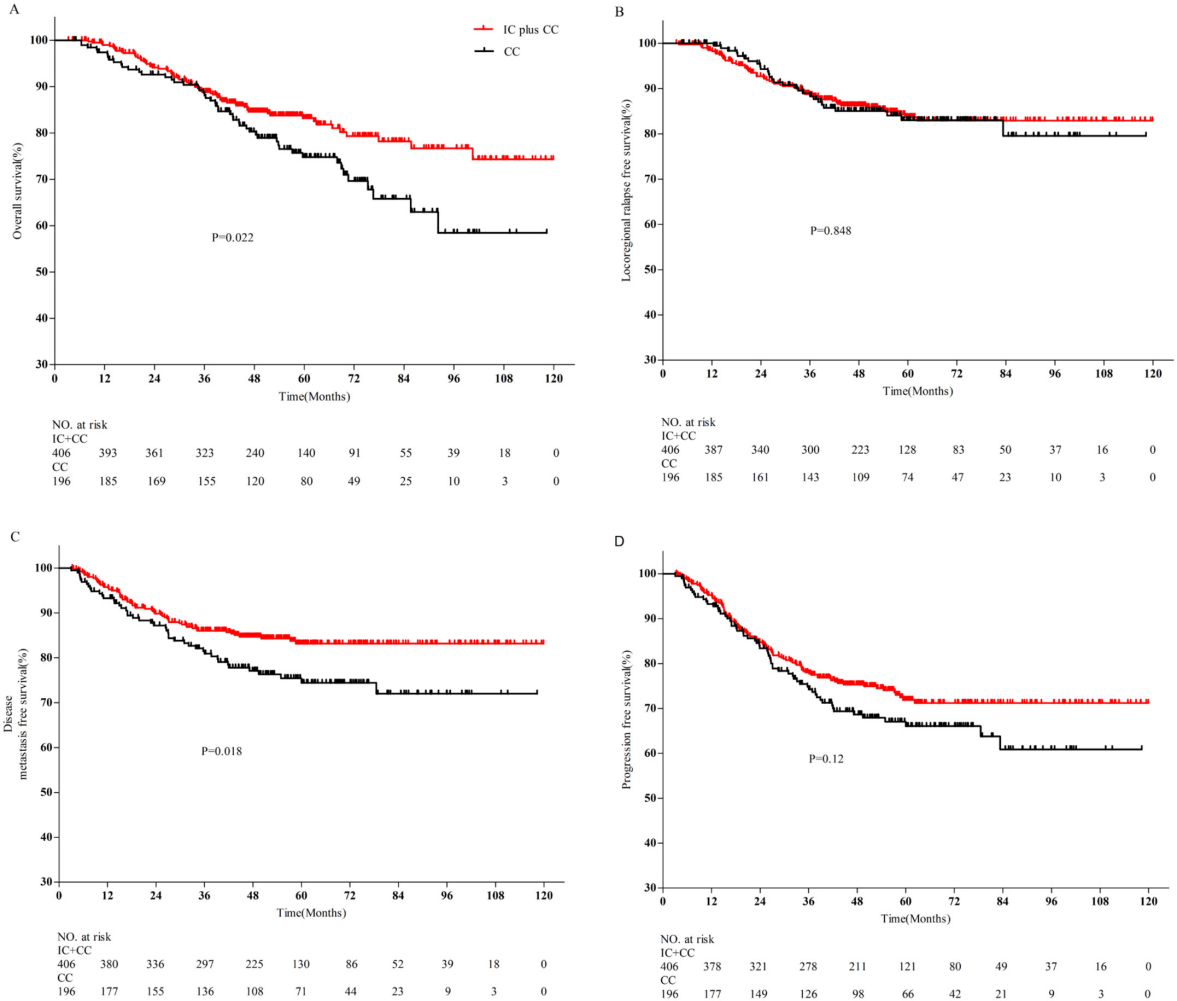
Kaplan-Meier survival curves for induction chemotherapy plus concurrent chemotherapy and concurrent chemotherapy alone in the 602 patients with stage IVa-b nasopharyngeal carcinoma treated with IMRT. **(A)** Overall survival; **(B)** locoregional relapse-free survival; **(C)** distant metastasis-free survival; **(D)** progression-free survival. Abbreviations: IMRT = intensity-modulated radiotherapy; IC = induction chemotherapy; CC = concurrent chemotherapy. *P*-values were calculated using the unadjusted log-rank test.

The median follow-up time was 52.3 months (range, 3.3–119.9 months). Table 3 summarizes the univariate analysis of baseline and clinical characteristics as prognostic factors (gender, age, WHO pathological type, T category, N category, chemotherapy strategy, clinical stage). Age, T category, N category, chemotherapy strategy and clinical stage were significantly associated with 5-year OS (*P* = 0.017, *P* = 0.031, *P* = 0.007, *P* = 0.022, and *P* = 0.001, respectively) in univariate analysis (Table 3). Moreover, patients with stage IVb NPC had significantly poorer prognoses in terms of 5-year DMFS and PFS than patients with stage IVa NPC (83.5% vs. 63.5, *P* < 0.001 and 72.1% vs. 59.5%, *P* = 0.002, respectively). In univariate analysis, the 5-year OS rate was 83.2% for induction chemotherapy plus concurrent chemotherapy and 74.8% for concurrent chemotherapy alone, with an absolute risk reduction of 8.4% (*P* = 0.022; Fig 1A). Induction chemotherapy plus concurrent chemotherapy and concurrent chemotherapy alone resulted in similar 5-year LRFS (83.7% vs. 83.0%, *P* = 0.848; Fig 1B) and 5-year PFS rates (71.9% vs. 66%, *P* = 0.12; Fig 1D). In addition, induction chemotherapy plus concurrent chemotherapy led to improved 5-year DMFS compared to concurrent chemotherapy alone (83.2.0% vs. 74.4%, *P* = 0.018; Fig 1C). Overall, patients treated with induction chemotherapy plus concurrent chemotherapy had a significantly better prognosis in terms of OS and DMFS compared to patients treated with concurrent chemotherapy alone.

**Table 3:** Summary of univariate analysis for prognostic factors in the 602 patients with stage IVa-b nasopharyngeal carcinoma.

Characteristic	*n*	5-year OS (%)	*P* *	5-year LRFS (%)	P*	5-year DMFS (%)	P*	5-year PFS (%)	P*
Gender			0.369		0.472		0.391		0.291
Male	472	79.1		82.3		79.6		68.8	
Female	130	84.2		87.0		82.7		74.3	
Age (years)			0.017		0.688		0.369		0.982
≤ 46	325	83.6		83.5		81.4		70.1	
> 46	277	76.1		83.3		79.0		70	
WHO pathological type			0.368		0.513		0.561		0.986
Type I	6	83.3		66.7		100		66.7	
Type II	32	89.5		80.9		79.5		70.3	
Type III	564	79.7		84.0		80.1		70	
T category			0.031		0.819		<0.001		0.017
T1	5	80		100		100		100	
T2	22	71.4		89.1		68.8		69.7	
T3	41	62.8		78.3		56.6		51.9	
T4	534	82.1		83.5		82.3		71.2	
N category			0.007		0.281		<0.001		0.001
N0	86	89.2		89		88		78.3	
N1	280	81.8		84.3		86.5		75.2	
N2	133	80.9		77.8		73.9		60.8	
N3	103	66.7		82.8		63.5		59.5	
Chemotherapy strategy			0.022		0.848		0.018		0.12
IC + CC	406	83.2		83.7		83.2		71.9	
CC	196	74.8		83.0		74.4		66.0	
Clinical stage			0.001		0.789		<0.001		0.002
IVa	499	83		83.6		83.5		72.1	
IVb	103	66.7		82.8		63.5		59.5	

Abbreviations: IC = induction chemotherapy; CC = concurrent chemotherapy; OS = overall survival; LRFS = locoregional relapse-free survival; DMFS = distant metastasis-free survival; PFS = progression-free survival; WHO = World Health Organization.

* *P*-values were calculated using the unadjusted log-rank test.

Multivariate analysis was performed to adjust for various prognostic factors. Gender, age, WHO pathological type, T category, N category, chemotherapy strategy and clinical stage were included as covariates. Consistent with the univariate analysis, the induction chemotherapy plus concurrent chemotherapy regimen was found to be an independent favorable prognostic factor for OS (HR, 0.62; 95% confidence interval [95% CI], 0.43–0.90, *P* = 0.012), DMFS (HR, 0.57; 95% CI, 0.38–0.83, *P* = 0.004; Table 4). Stage IVb NPC was associated with an increased risk of death compared to stage IVa NPC (HR, 2.13; 95% CI, 1.40–3.25, *P* < 0.001). Moreover, advanced N category was associated with an increased risk of distant metastasis and disease progression in the entire cohort (Table 4).

**Table 4:** Summary of multivariate analysis of prognostic factors in the 602 patients with stage IVa-b nasopharyngeal carcinoma.

	Characteristic	HR	95% CI	*P*-value*
OS	Age > 46 years	1.56	1.08–2.24	0.017
	Stage IVb	2.13	1.40–3.25	<0.001
	IC + CC	0.62	0.43–0.90	0.012
LRFS	……	……	……	……
DMFS	N category	1.73	1.41–2.11	<0.001
	IC + CC	0.57	0.38–0.83	0.004
PFS	N category	1.37	1.16–1.61	<0.001

Abbreviations: IC = induction chemotherapy; CC = concurrent chemotherapy; OS = overall survival; LRFS = locoregional relapse-free survival; DMFS = distant metastasis-free survival; PFS = progression-free survival; HR = hazard ratio; CI = confidence interval.

* *P*-values were calculated using an adjusted Cox proportional hazards model with the forward conditional method.

We performed subgroup analysis for the patients with stage IVa and IVb NPC. Induction chemotherapy plus concurrent chemotherapy did not provide a significant benefit over concurrent chemotherapy in terms of 5-year OS (*P* = 0.055), 5-year LRFS (*P* = 0.789) or 5-year PFS (*P* = 0.077) in patients with stage IVa NPC. However, the addition of induction chemotherapy significantly improved DMFS by 9.5% (86.8% vs. 77.3%, *P* = 0.008; Table 5) in patients with stage IVa NPC. In the stage IVb subgroup, induction chemotherapy plus concurrent chemotherapy did not significantly improve 5-year OS, LRFS, DMFS or PFS (*P* = 0.059, *P* = 0.926, *P* = 0.336, *P* = 0.622, respectively) compared to concurrent chemotherapy alone (Table 5).

**Table 5:** Summary of subgroup analysis of the effect of induction chemotherapy in patients with stage IVa and IVb nasopharyngeal carcinoma.

	*n*	5-year OS (%)	P*	5-year LRFS (%)	P*	5-year DMFS (%)	P*	5-year PFS (%)	P*
Stage IVa			0.055		0.789		0.008		0.077
IC + CC	329	85.8		83.7		86.8		74.3	
CC	170	78		83.3		77.3		67.7	
Stage IVb			0.059		0.926		0.336		0.622
IC+CC	77	71.0		84.3		67.1		61.7	
CC	26	54.5		79.1		52.9		53.1	

Abbreviations: IC = induction chemotherapy; CC = concurrent chemotherapy; OS = overall survival; LRFS = locoregional relapse-free survival; DMFS = distant metastasis-free survival; PFS = progression-free survival.

* *P*-values were calculated using the unadjusted log-rank test.

## Discussion

Although controversy regarding the evidence supporting the administration of induction chemotherapy in LA-NPC exists, induction chemotherapy offers a number of advantages that could be beneficial under certain conditions. Lee et al. [21] reported induction chemotherapy using cisplatin and 5-fluorouracil shrank the primary tumor or even significantly down-staged the T category (*P* = 0.016), resulting in a wider margin for irradiation and better protection of important normal tissues such as critical neurological structures. Moreover, these improvements in radiation dose coverage effectively eradicated micro-metastases to lower the risk of metastases. In previous clinical trials, induction chemotherapy regimens such as cisplatin plus docetaxel [13], gemcitabine plus carboplatin, paclitaxel [15], and cisplatin plus fluorouracil [22] have demonstrated tolerable toxicities. However, induction chemotherapy may adversely affect patient tolerance to concurrent chemoradiotherapy. In the phase 2/3 trial by Tan et al. [15], the induction chemotherapy group had a higher frequency of grade 3 and 4 toxicities such as leukopenia, neutropenia, thrombocytopenia compared with the chemoradiotherapy alone group; these toxicities often resulted in patients experiencing dose reductions during concurrent chemotherapy, which compromised the therapeutic efficacy. Therefore, it is imperative to be able to identify high risk patients who may benefit significantly from induction chemotherapy.

In a previous trial of LA-NPC, Fountzilas et al. [14] observed no significant differences in 3-year OS (66.6% vs. 71.8%, *P* = 0.888) and 3-year PFS (64.5% vs. 63.5%, *P* = 0.334) between the induction chemotherapy plus concurrent chemotherapy group and concurrent chemotherapy group. Tan et al. obtained similar results [15], even though nearly all of the patients underwent IMRT (94.3% vs. 92.3% for 3-year OS, 83.8% vs. 79.9% for 3-year DMFS, 74.9% vs. 67.4% for 3-year PFS, respectively). One critical factor that may explain these negative results is the possibility that induction chemotherapy may only be of benefit in certain patients with a high risk of metastasis. Therefore, it is important to be able to identify high risk patients to explore the value of additional induction chemotherapy in these subgroups. In addition, whether a 3-year median follow-up time is sufficient to adequately assess the benefits of induction chemotherapy should be taken in consideration.

Patients with stage IVa-b NPC have high T categories (T4) or bulky cervical nodal metastases (N3), which can be negative prognostic factors for survival. Tumors with intracranial extension and/or involvement of the cranial nerves, which are classified as T4, are considered to have a poor prognosis [23]. Moreover, studies of other head and neck cancers have demonstrated perineural tumor spread is associated with a higher incidence of distant metastases [24]. In 2011, the National Comprehensive Cancer Network (NCCN) recommended chemoradiotherapy plus adjuvant or induction chemotherapy as the standard treatment regimen for T4 NPC. However, few clinical trials have focused on whether patients with stage IVa-b NPC could benefit from induction chemotherapy before chemoradiotherapy. In our retrospective study, all 602 patients with stage IVa-b NPC accepted IMRT and the median follow-up time was nearly 5 years. As stated previously, the addition of induction chemotherapy significantly improved 5-year OS and DMFS (*P* = 0.022 and *P* = 0.018, respectively) compared with concurrent chemotherapy alone. This positive result provides further evidence for the necessity of induction chemotherapy regimens in LA-NPC. The use of IMRT may have led to better disease control compared to conventional techniques, which may partially explain why no significant differences in 5-year LRFS (83.7.0% vs. 83.0%, *P* = 0.848) and 5-year PFS (71.9% vs. 66.0%, *P* = 0.12) were observed between the induction chemotherapy plus concurrent chemotherapy and concurrent chemotherapy groups in this study. Stage IVb and advanced N category were independent unfavorable prognostic factors for overall survival, disease progression and distant metastasis, which is consistent with the high risk we defined.

In subgroup analysis, induction chemotherapy plus concurrent chemotherapy did not significantly improve 5-year OS in the IVa or IVb subgroups, but did marginally increase overall survival in both subgroups (*P* = 0.055, *P* = 0.059, respectively). We suggest the small sample sizes in the subgroup analysis may have introduced bias to some extent. With respect to 5-year DMFS, and as previously mentioned, high T category or bulky cervical nodal metastases may help to explain why induction chemotherapy reduces distant metastasis in stage IVa NPC. However, there is little scope for the addition of induction chemotherapy to improve 5-year DMFS in stage IVb NPC for several reasons. Firstly, patients with N3 NPC can have both bulky cervical nodal metastases (maximal diameter > 6 cm) and supraclavicular fossa nodal metastases [17], which are associated with poor prognosis. Micro-metastases may have already spread to other distant organs or tissues; it remains unclear how effectively induction chemotherapy can target these undetectable lesions. Secondly, the patients in this study received a maximum of three induction chemotherapy cycles; whether an increased number of induction chemotherapy cycles is required control advanced N category NPC warrants further exploration. Thirdly, the subgroups were relatively small: the stage IVb NPC concurrent chemotherapy group only included 26 patients. Fourthly, as this was a retrospective analysis, it is inevitable that oncologists were more likely to prescribe induction chemotherapy plus concurrent chemotherapy to patients with LA-NPC who originally had a poor prognosis.

A trial in Singapore suggested induction chemotherapy was of benefit in patients with detectable baseline plasma EBV DNA [25]. Nevertheless, we did not consider Epstein-Barr virus (EBV) DNA copy number or EBV antibody titers in this analysis, which is a limitation of this study. No survival benefits were observed for induction chemotherapy in an unselected cohort of patients with LA-NPC in the trial by Tan [15], further indicating the importance of more comprehensive analysis of the value of induction chemotherapy in specific high risk subgroups of patients with LA-NPC.

## Conclusion

This retrospective study indicates the addition of induction chemotherapy to concurrent chemotherapy provides a benefit in terms of 5-year OS and 5-year DMFS in patients with stage IVa-b NPC treated with IMRT. The results of this study have significant implications for the management of LA-NPC. Appropriate methods of selecting high risk patients should be considered and tested in clinical therapeutic trials.

## Supporting Information

S1 File Primary data for each analysis. (XLS)
